# Tuberculosis knowledge and attitude among non-health science university students needs attention: a cross-sectional study in three Ethiopian universities

**DOI:** 10.1186/s12889-020-08788-1

**Published:** 2020-05-06

**Authors:** Abiyu Mekonnen, Jeffery M. Collins, Eveline Klinkenberg, Dawit Assefa, Abraham Aseffa, Gobena Ameni, Beyene Petros

**Affiliations:** 1 Department of Medical Laboratory Sciences, Menelik-II College of Medical and Health Sciences, Kotebe Metropolitan University, Po-Box: 11422, Addis Ababa, Ethiopia; 2 Division of Infectious Diseases, Department of Medicine, Emory University School of Medicine, Atlanta, GA USA; 3 Technical division, KNCV Tuberculosis Foundation, the Hague, the Netherlands/ Department of Global Health and Amsterdam Institute for Global Health and Development, Amsterdam University Medical Centers, location Academic Medical Center, Amsterdam, the Netherlands; 4 KNCV Tuberculosis Foundation, Addis Ababa, Ethiopia; 5 Armauer Hansen Research Institute, Addis Ababa, Ethiopia; 6 Aklilu Lemma Institute of Pathobiology, Addis Ababa University, Addis Ababa, Ethiopia; 7 Department of Microbial, Cellular and Molecular Biology, Addis Ababa University, Addis Ababa, Ethiopia

**Keywords:** TB, Knowledge and attitude, University students, Ethiopia

## Abstract

**Background:**

Ethiopia is among the 14 high TB, TB/HIV and MDR-TB burden countries globally. Prior studies indicate students attending universities in Ethiopia may be at increased risk for active tuberculosis (TB) relative to the general population, mainly due to the dramatic increase in expansion of the enrollment scale of universities.This study sought to gain insight about non-health science university students’ TB knowledge and attitudes to help develop a strategy for TB education in this population.

**Methods:**

A cross-sectional study was conducted from October to December 2018 among non-health science university students at three eastern Ethiopia public universities. Participants were considered having ‘good’ knowledge on TB when they correctly mentioned the communicability, means of transmission and prevention methods of TB and recognized modern medicine as the best treatment for TB. Participants were considered as having ‘acceptable’ attitude towards TB when they indicated they would seek immediate care for TB diagnosis, not hide a TB diagnosis and feel compassion to help people with TB.

**Results:**

A total of 1720 non-health science university students participated. Only 614 (35.7%) of the students had ‘good’ knowledge on TB. This differed significantly between universities, with students from Haramaya and Dire Dawa universities more likely to have ‘good’ TB knowledge than their counterparts from Jigjiga University [COR (Crude Odds Ratio):1.62 and 1.94, respectively; and 95% Confidence Interval (CI): (1.236, 2.079) and (1.511, 2.483), respectively]. Only a third of students, 555 (32.3%) mentioned ‘bacteria’ as causing TB, and 836 students (48.6%) had ever heard of Multi Drug Resistant-TB (MDR-TB). An ‘acceptable’ attitude towards people with TB was observed in 666 students (38.7%). Even though 739 students (43%) felt compassion and desire to help TB patients, 213 (12%) and 382 (22%) mentioned they fear and tend to stay away from TB patients, respectively.

**Conclusions:**

The present study revealed that non-health science university students lack important TB knowledge and have misconceptions about TB in eastern Ethiopia. University administrators and other stakeholders striving against TB should provide due attention to university settings and consider development of student education programs to improve awareness and knowledge of TB disease.

## Background

Tuberculosis (TB) is the leading cause of infectious disease mortality, resulting in an estimated 1.5 million deaths globally each year [1]. Ethiopia is one of the fourteen countries with the highest burden of TB, TB/HIV and MDR-TB cases with an estimated incidence rate of 151/100,000 population in 2018 [1]. However, despite the high burden of disease, studies conducted to assess TB knowledge and attitudes in the general population of Ethiopia indicate that there are misconceptions about TB and many believe it is an incurable and an extremely dangerous disease [2, 3].

Low levels of TB awareness in a specific communities lead to stigma and discrimination of those affected by the disease [2], while those with lower levels of knowledge and negative attitudes demonstrate inefficient use of health care and poor disease prevention behavior [4, 5]. Therefore, characterizing the level of current knowledge about TB in different populations is important to identify misconceptions and determine which groups would benefit from interventions to improve awareness [6].

College students in Ethiopia have been identified as having an increased risk of TB disease. A previous study in Ethiopian universities revealed a high incidence of pulmonary TB (PTB) with an estimated 433 cases per 100,000 student population [7]. One potential cause for this increased incidence is overcrowding on university campuses [7, 8]. The number of public universities increased from 5 to 45 in less than two decades while enrollment increased from 27,000 in 2003/04 academic year into 380,000 in 2015/16 academic year [9]. However, little is known about the knowledge and attitudes of the student population towards persons with TB disease and whether this may contribute to the higher TB incidence observed in this segment of the population.

To our knowledge, no previous study has assessed the knowledge of and attitudes towards TB among university students in Ethiopia. We sought to investigate TB knowledge and attitudes among Ethiopian university students to determine whether deficits in knowledge and negative attitudes could play a role in the higher TB incidence observed in this population. We focused on non-health science students at three public universities in eastern Ethiopia previously found to have a high TB incidence relative to the general population. Our goal was to better understand TB awareness among non-health science students and identify gaps in knowledge to guide future educational interventions.

## Methods

### Study setting

The study was conducted among non-health science students of the three universities located in the eastern part of Ethiopia: Haramaya University located in Oromia Regional State, Dire Dawa University located in Dire Dawa city administration and Jigjiga University located in Somali Regional State. The three universities studied were selected because prior estimates of TB incidence among the student population of each campus indicate a significantly higher TB incidence rate relative to the general population [7]. Furthermore, the eastern part of Ethiopia, where the three universities are located, is well known for its high TB burden compared with the rest of the country [10].

### Study design and sampling technique

A cross-sectional study was conducted to assess the level of knowledge and attitude towards TB among non-health science students attending one of the three selected universities. We used stratified random sampling with a proportional allocation technique to select the sample of students from the three universities studied. The sample size calculated was allocated proportional to the number of students living in each dormitory building for both male and female students. Students from each building were randomly selected for inclusion in the study. Surveys were conducted from October to December 2018.

### Sample size determination

The overall full time student population for the universities combined was about 38,315, (15,875 for Haramaya University (HU), 11,402 for Dire Dawa University (DDU), and 11,038 for Jigjiga University (JJU). Assuming random distribution of TB knowledge among university students, and 4% margin of error tolerated, the following formula was used to determine the sample size for each university as described previously [11].

n = N/1 + Nd^2^.

Where n = sample size required, N = population size, d = margin of error (d = 4%), and with 95% Confidence Interval the sample size for each university was: 579 (HU), 571 (DDU), and 570 (JJU).

### Questionnaire and interviewing

Interviews were conducted over a 30–40 min period using a standardized questionnaire designed for TB knowledge and attitude assessment [12], with some amendment following WHO guidance [13]. The original questionnaire was validated in a population of prison inmates and had nine socio-demographic questions, nine TB knowledge questions, and ten TB attitude questions. The survey was pre-tested and amended to more appropriately fit the context of university students. The socio-demographic section was shortened to six questions while the knowledge and attitude assessments were expanded to 20 and 14 questions, respectively (see Additional file-3 for the complete questionnaire). Data were collected by trained nurses and public health officers under close supervision from the principal investigator of the study.

Categorization of study participants as having ‘good’ knowledge or ‘poor’ knowledge and having ‘acceptable’ attitude or ‘unacceptable’ attitude was as described previously [12] with some amendments. Students who mentioned all the following four items were considered to have ‘good’ knowledge: TB is a communicable disease, TB is transmitted through the air when an infected person coughs or sneezes, TB can be prevented by covering mouth and nose when coughing and sneezing, and modern medicine/specific drugs given at health facilities is the best treatment for TB. However, those who missed one or more of the four items were categorized as having ‘poor’ knowledge on TB. Whereas, the following three elements were used to categorize study participants’ attitudes: students who indicated they would seek immediate medical care if they were diagnosed with TB, students who stated that they would not hide their diagnosis if they had TB and students who indicated they feel compassion and a desire to help people with TB. Students who responded affirmative to those three elements were categorized as having an ‘acceptable’ attitude towards TB patients and those who answered ‘no’ to one or more items were considered to have an ‘unacceptable’ attitude towards TB patients.

### Data analysis

Data were entered in to EpiData version 3.1 and exported to SPSS Version 23 (IBM, Armonk, NY, USA) for statistical analysis. The frequency and proportion of different variables were reported by descriptive statistics. The association between participant characteristics and their knowledge and attitude towards tuberculosis was assessed using univariate logistic regression model with Odds Ratio (OR) and 95% Confidence Interval (CI). All variables with *P* < 0.25 in the univariate analysis were included in the multivariate logistic regression model. A *p*-value < 0.05 was considered statistically significant.

## Results

A total of 1720 students from the three public universities in eastern Ethiopia agreed to participate in the study: 579 (33.7%) from Haramaya University, 571 (33.2%) from Dire Dawa University and 570 (33.1%) from Jigjiga University. In all the three universities, only about 10 students refuse to participate in the study, and all cited competing obligations as the reason for not participating and were substituted by other willing students. The majority of the participants were male (1194, 69.4%) and rural residents (1006, 58.5%). The highest number of students came from Oromia regional state (717, 41.7%), followed by Amhara (417, 24.2%), Southern Nation and Nationalities People (SNNP) (163, 9.5%) and Somali (150, 8.7%) regional states (Table 1).

**Table 1:** Socio-demographic characteristics of study participants by university, eastern Ethiopia

Variable	University		
	Harmaya University	Dire Dawa University	Jigjiga University
**Gender**			
Male	413 (71.3)	394 (69.0)	387 (67.9)
Female	166 (28.7)	177 (31.0)	183 (32.1)
**Age group**			
18–20	258 (44.6)	306 (53.6)	313 (54.9)
21–23	285 (49.2)	235 (41.2)	206 (36.1)
24+	36 (6.2)	30 (5.2)	51 (8.9)
**Region of origin before enrolling in university**			
Tigray	13 (2.2)	12 (2.1)	18 (3.2)
Amhara	120 (20.7)	152 (26.6)	145 (25.4)
Oromia	306 (52.8)	234 (41.0)	177 (31.1)
Somali	24 (4.1)	24 (4.2)	102 (17.9)
SNNP*	49 (8.5)	53 (9.3)	61 (10.7)
Addis Ababa	34 (5.9)	53 (9.3)	31 (5.4)
Dire Dawa	14 (2.4)	33 (5.8)	20 (3.5)
Others	19 (3.3)	10 (1.8)	16 (2.8)
**Type of Residence before enrolling in university**			
Urban	273 (47.2)	224 (39.2)	222 (38.9)
Rural	306 (52.8)	347 (60.8)	348 (61.1)
**Source of information about TB**			
Radio	280 (48.4)	306 (53.6)	237 (41.6)
Friends	70 (12.1)	54 (9.5)	55 (9.6)
Health care center	129 (22.3)	96 (16.8)	174 (30.5)
Television	50 (8.6)	82 (14.4)	65 (11.4)
Others	50 (8.6)	33 (5.8)	39 (6.8)

* SNNP: Southern Nations and Nationality People

### Knowledge about cause, transmission, treatment and prevention of tuberculosis

The vast majority of the study participants knew that TB is a communicable disease and its mode of transmission is through the air when an infected person coughs or sneezes (1390, 80.8% and 1439, 83.7%, respectively; Additional file 1, Table 2). However, only 555 (32.3%) knew that a bacterium is the cause of TB disease. There were 1414 (82.2%) persons who mentioned ‘modern medicine’ is the best means of treatment for TB disease, though only 583 (41.2%) had heard of the Directly Observed Treatment, Short-course (DOTS) program. The majority of students (1008, 58.6%) were aware that ‘covering mouth and nose when coughing or sneezing’ can prevent TB transmission. Only 836 (48.6%) and 180 (10.5%) students mentioned that they have heard of MDR-TB and latent TB, respectively (Additional file 1, Table 2).

### Factors associated with greater TB knowledge

The majority of the study participants (1106, 64.3%) had ‘poor’ knowledge about tuberculosis. Out of the four elements used for categorization of the level of TB knowledge of study participants, 519 (30.2%), 352 (20.5%) and 155 (9%) were knowledgeable in three, two and one out of the four elements, respectively. There was a statistically significant difference between universities with students from Haramaya and Dire Dawa universities more likely to demonstrate ‘good’ TB knowledge than those from Jigjiga University [Crude Odds Ratio (COR) 1.62 and 1.94 and 95% Confidence Interval (CI) of (1.263, 2.079) and (1.511, 2.483), respectively (Table 3)].

**Table 3:** Tuberculosis knowledge and its association with participants’ demography among university students, eastern Ethiopia

Variable	Knowledge		COR^**#**^ (95% CI)	AOR^¥^ (95%CI)
	Poor n (%)	Good n (%)		
All participants	1106 (64.3)	614 (35.7)		
**University**				
Haramaya University	360(62.2)	219 (37.8)	1.62 (1.263, 2.079)	1.59 (1.204, 2.093)
Dire Dawa University	331 (58%)	240 (42.0)	1.94 (1.511, 2.483)	2.07 (1.570, 2.717)
Jigjiga University	415 (72.8)	155 (27.2)	1	
**Gender**				
Male	769 (64.4)	425 (35.6)	0.99 (0.796, 1.220)	NA
Female	337 (64.1)	189 (35.9)	1	
**Age group**				
18–20	598 (68.2)	279 (31.8)	0.70 (0.468, 1.032)	NA
21–23	438 (60.3)	288 (39.7)	0.98 (0.658, 1.459)	NA
24+	70 (59.8)	47 (40.2)	1	
**Region of origin before enrolling in university**				
Tigray	23 (53.5)	20 (46.5)	1.58 (0.670, 3.707)	2.39 (0.570, 10.02)
Amhara	266 (63.8)	151 (36.2)	1.03 (0.541, 1.956)	1.77 (0.474, 6.582)
Oromia	460 (64.2)	257 (35.8)	1.01(0.540, 1.900)	1.69 (0.456, 6.266)
Somali	115 (76.7)	35 (23.3)	0.55 (0.269, 1.131)	1.11(0.284, 4.337)
SNNP*	109(66.9)	54 (33.1)	0.90 (0.449, 1.794)	1.47 (0.384, 5.652)
Addis Ababa	68 (57.6)	50 (42.4)	1.33 (0.654, 2.714)	2.21 (0.571, 8.542)
Dire Dawa	36 (53.7)	31 (46.3)	1.56 (0.718, 3.393)	2.72 (0.676, 10.92)
Others	29 (64.4)	16 (35.6)	1	1
**Type of Residence before enrolling in university**				
Urban	430 (59.8)	289 (40.2)	1.40 (1.145, 1.706)	1.47 (1.191, 1.824)
Rural	676 (67.5)	325 (32.5)	1	1
**Year of student in the university**				
Year-1	275 (69.3)	122 (30.7)	1	1
Year-2	220 (67.3)	107 (32.7)	1.10 (0.801, 1.501)	1.07 (0.760, 1.509)
Year-3	336 (67.3)	163 (32.7)	1.10 (0.823, 1.452)	1.06 (0.777, 1.450)
Year-4	175 (57.4)	130 (42.6)	1.67 (1.226, 2.286)	1.77 (1.233, 2.537)
Year-5	76 (56.3)	59 (43.7)	1.75 (1.171, 2.614)	1.63 (1.062, 2.497)
Year-6	24 (42.1)	33 (57.9)	3.10 (1.76, 5.466)	3.41 (1.894, 6.143)
**Source of information about TB**				
Radio	489 (59.4)	334 (40.6)	1.45 (0.969, 2.180)	1.49 (0.971, 2.286)
Friends	121 (67.6)	58 (32.4)	1.02 (0.623, 1.670)	1.08 (0.642, 1.822)
Health care center	277 (69.4)	122 (30.6)	0.94 (0.606, 1.450)	1.02 (0.646, 1.618)
Television	136 (69.0)	61 (31.0)	0.96 (0.587, 1.552)	0.98 (0.584, 1.656)
Others	83 (68.0)	39 (32.0)	1	

* SNNP: Southern Nations and Nationality People; NA: Not applicable; ^#^COR: Crude Odds Ratio; ^¥^AOR: Adjusted Odds Ratio

There was also an association between participants’ level of TB knowledge and their community environment before joining the university. Students from an urban area were 1.4 times more knowledgeable than their rural counterparts (95% CI: 1.145, 1.706). Students who had spent more time in the universities were more knowledgeable than students in the first three years (Table 3). All those variables statistically significant in the univariate analysis remained significant in the multivariate regression model (Table 3).

### Attitudes towards tuberculosis

The majority of study participants (1049, 61.0%) said that, if they had been diagnosed with TB, they would seek immediate medication, but a considerable number stated that they would feel fear (294, 17.1%) or sadness and hopelessness (142, 8.3%). Three hundred forty-four (20%) of the participants said that they would not disclose their diagnosis if they had TB, and 555 (32.3%) perceived that, if they had TB, it would affect their social relationships. Many participants (229, 12.9%) believe TB treatment is expensive. Even though a considerable number of students indicated to feel compassion and a desire to help TB patients (739, 43%), 213 (12%) fear TB patients and 382 (22%) tend to stay away from them (Additional file 2, Table 4).

### Factors associated with unacceptable attitude towards TB

Only 666 (38.7%) had an ‘acceptable’ attitude towards tuberculosis patients. Univariate analysis showed that, similar to TB knowledge, there was a statistically significant difference in students’ attitude towards tuberculosis patients by the university where they are attending their courses. Students in Haramaya and Dire Dawa Universities were nearly two times more likely to have an ‘acceptable’ attitude towards tuberculosis cases compared with their counterparts in Jigjiga University, [Adjusted Odds Ratio (AOR): 1.86 and 1.42, and 95%CI of (1.433, 2.421) and (1.091, 1.848), respectively]. The other variables did not show any statistically significant difference in students’ attitude towards tuberculosis (Table 5).

**Table 5:** Participants attitude towards TB patients and its association with demography among university students, eastern Ethiopia

Variable	Attitude towards TB patients		COR^**#**^ (95% CI)	AOR^**¥**^ (95%CI)
	Unacceptable n (%)	Acceptable n (%)		
**All participants**	1054 (61.3)	666 (38.7)		
**University**				
Haramaya University	313 (54.1)	266 (45.9)	2.05 (1.608, 2.616)	1.86 (1.433, 2.421)
Dire Dawa University	338 (59.2)	233 (40.8)	1.66 (1.301, 2.127)	1.42 (1.091, 1.848)
Jigjiga University	403 (70.7)	167 (29.3)	1	1
**Gender**				
Male	742 (62.1)	452 (37.9)	0.89 (0.720, 1.095)	NA
Female	312 (59.3)	214 (40.7)	1	
**Age group**				
18–20	553 (63.1)	324 (36.9)	0.71 (0.480, 1.044)	0.66 (0.433, 1.004)
21–23	437 (60.2)	289 (39.8)	0.80 (0.539, 1.183)	0.82 (0.541, 1.246)
24+	64 (54.7)	53 (45.3)	1	1
**Region of origin before enrolling in university**				
Tigray	25 (58.1)	18 (41.9)	1.98 (0.808, 4.853)	2.20 (0.869, 5.540)
Amhara	247 (59.2)	170 (40.8)	1.89 (0.950, 3.770)	2.07 (1.013, 4.242)
Oromia	425 (59.3)	292 (40.7)	1.89 (0.960, 3.719)	1.75 (0.868, 3.534)
Somali	96 (64.0)	54 (36.0)	1.55 (0.738, 3.242)	1.88 (0.863, 4.083)
SNNP*	108 (66.3)	55 (33.7)	1.40 (0.671, 2.924)	1.42 (0.665, 3.049)
Addis Ababa	80 (67.8)	38 (32.2)	1.31 (0.608, 2.808)	1.33 (0.602, 2.915)
Dire Dawa	40 (59.7)	27 (40.3)	1.86 (0.816, 4.221)	1.76 (0.754, 4.111)
Others	33 (73.3)	12 (26.7)	1	
**Type of Residence before enrolling in university**				
Urban	437 (60.8)	282 (39.2)	1.04 (0.852, 1.262)	NA
Rural	617 (61.6)	384 (38.4)	1	
**Year of student in the university**				
Year-1	227 (57.2)	170 (42.8)	1.03 (0.587, 1.807)	1.10 (0.615, 1.962)
Year-2	213 (65.1)	114 (34.9)	0.74 (0.415, 1.305)	0.75 (0.414, 1.365)
Year-3	327 (65.5)	172 (34.50	0.72 (0.414, 1.263)	0.69 (0.387, 1.235)
Year-4	189 (62.0)	116 (38.0)	0.84 (0.475, 1.499)	0.90 (0.492, 1.659)
Year-5	65 (48.1)	70 (51.9)	1.48 (0.793, 2.766)	1.49 (0.777, 2.857)
Year-6	33 (57.9)	24 (42.1)	1	1
**Source of information about TB**				
Radio	502 (61.0)	321 (39.0)	0.99 (0.668, 1.455)	NA
Friends	98 (54.7)	81 (45.3)	1.27 (0.798, 2.034)	NA
Health care center	262 (65.7)	137 (34.3)	0.81 (0.531, 1.224)	NA
Television	118 (59.9)	79 (40.1)	1.03 (0.650, 1.638)	NA
Others	74 (60.7)	48 (39.3)	1	

* SNNP: Southern Nations and Nationality People; NA: Not applicable; ^#^COR: Crude Odds Ratio; ^¥^AOR: Adjusted Odds Ratio

### Association between tuberculosis knowledge and attitudes

Students with ‘good’ TB knowledge were significantly more likely than those with ‘poor’ knowledge to seek immediate medical care and not hide their diagnosis if diagnosed with TB and to have positive feelings for people with TB disease [Crude Odds Ratio (COR) (95%CI) 1.82 (1.474, 2.241)], [COR (95%CI) 2.34 (1.860, 2.934)] and [COR (95%CI) 2.09 (1.707, 2.550), respectively] (Table 6). Seventy percent and 56% of the students with ‘good’ and ‘poor’ TB knowledge respectively felt to seek immediate medication if he/she is diagnosed with TB (Table 6).

**Table 6:** Tuberculosis knowledge and its association with attitude towards tuberculosis among university students, eastern Ethiopia

Variable	Tuberculosis Knowledge		COR* (95% CI)
	Poor n (%)	Good n (%)	
**Feeling if he/she is diagnosed with TB**			
Don’t seek immediate medication	486 (43.9)	185 (30.0)	
Seek immediate medication	620 (56.1)	429 (70.0)	1.82 (1.474, 2.241)
**Would you hide it if you had TB?**			
Hide	429 (76.6)	131 (23.4)	1
Not hide	677 (58.4)	483 (41.6)	2.34 (1.860, 2.934)
**Perception about TB affecting his/her social relationship, if he/she is infected**			
No	562 (64.7)	306 (35.3)	1
Yes	343 (61.8)	212 (38.2)	1.14 (0.910, 1.415)
Do not know	201 (67.7)	96 (32.3)	0.88 (0.663, 1.161)
**Feeling about people with TB disease**			
Negative feeling	702 (71.6)	279 (28.4)	1
Positive feeling	404 (54.7)	335 (45.3)	2.09 (1.707, 2.550)

*COR: Crude Odds Ratio

## **Discussio**n

In the present study, we found that only about one third of Ethiopian university students had a ‘good’ level of TB knowledge and that most students had at least some ‘unacceptable’ attitudes towards TB. These findings suggest that despite the high burden of TB disease observed in Ethiopian universities [7, 8] there remains a need for educational interventions to improve TB knowledge and attitudes among students. Improving student knowledge and attitudes towards TB will therefore need to be an important part of new programs focusing on improving TB diagnosis and reducing TB transmission on university campuses [14].

The vast majority of students know that TB is an infectious disease which is transmissible, although the majority doesn’t know its cause is a bacterium. Knowing TB is caused by bacteria would enable students adhere to proper infection prevention and treatment strategies. More than one in four students mentioned ‘cold wind’ as cause of TB, which is a reflection of common misconception in Ethiopia that TB is an illness caused by the cold or blowing wind and categorized as a ‘bird’ [15]. University students are generally better educated than other Ethiopians and are assumed to have better knowledge regarding TB transmission, treatment and drug resistance. Therefore, the observed critical misconceptions about the cause of the disease could by itself be a major barrier to appropriate care-seeking behaviors [16, 17].

Even though the vast majority considered modern medicine/specific drugs given at health facilities as the best treatment for TB, only about one in three students knew about the DOTS program, despite it having been implemented in Ethiopia over 20 years ago. In addition, less than 10% of the students mentioned development of drug resistance as a risk if someone does not complete anti-TB treatment and about half were not familiar with MDR-TB. In line with our findings, studies among non-medical students also showed poor knowledge about latent TB, the DOTS program [11] and MDR-TB [18]. These findings may indicate an overall poor knowledge about TB treatment in the student community.

The present study also revealed that the level of knowledge and attitudes of students about TB varied with the higher education institution attended. Ten percent more students from Jigjiga University had ‘poor’ TB knowledge compared to the other two universities. A prior study found the odds of having PTB were about two times higher among students in Jigjiga University versus students at Haramaya University [7]. This suggests that less TB knowledge could be contributing to a higher burden of TB on campus and highlights the need for a health education program for students in Jigjiga University in particular.

Students that originated from rural areas were less knowledgeable than their urban counterparts. This finding is in line with a previous study from northern Ethiopia [2], where individuals from rural areas had a significantly lower level of TB knowledge than urban residents. Possible explanations for this finding include lower health literacy and poor access to health information and healthcare services in rural areas [2]. A previous study from a pastoralist rural community of Somali regional state (the region where the present study was conducted) also reported poor knowledge and limited access to health facilities, which led to long delays before seeking TB treatment [19].

Students who had spent more years in the university were more knowledgeable about TB than students in the first three years. This suggests that students gain more knowledge about TB disease after spending more time attending university. A previous study in the same setting [7], showed that the prevalence of PTB is higher among students who have been on university campuses for over 2 years. It is therefore possible that students in later years are more likely to have had personal experience with TB or know someone diagnosed with TB, leading to greater knowledge and fewer negative attitudes.

The majority of the study participants had ‘unacceptable’ attitudes towards TB patients, which could lead to stigma and discrimination of students affected by the disease and have a further negative impact on care-seeking behavior [2]. This stigma may be related, in part, to HIV as more than two in three students related TB to people with HIV.

Only slightly more than a quarter of students stated that their home community supports and helps TB patients. The remainder were either not sure what to say or felt their community has misconception regarding TB. This finding is in agreement with misconceptions of the general population about TB patients elsewhere in Ethiopia [2, 3]. This unacceptable attitude in the student community towards TB patients and TB disease also indicates efforts are needed to create proper awareness of TB and change attitudes towards TB both on university campuses and in the larger society.

This study also shows effective education and awareness campaigns could be delivered to students on a variety of platforms. The majority of participants mentioned ‘Radio’ as key source of information about TB, which could be one of the possible justifications for the ‘good knowledge’ documented in this study when individual answers are analyzed. Thus, university or surrounding radio stations could be further strengthened to serve as an effective way to communicate TB-related information to university students. Hanging TB posters or advertising on university campuses and surrounding towns to expose students to information about TB are also considerations. Dedicated educational programs and campus meetings targeting students may have a role as well. Regional health bureaus could work together with the university administration and local health facilities to facilitate the dissemination of information via these various modalities.

This study is subject to limitations. Participants were asked specific questions about TB knowledge and attitudes, which may not fully capture the knowledge and beliefs held by some students. It is possible that some students may have been able to identify correct answers based on the format of the questionnaire that do not actually reflect their true level of knowledge. We have categorized students as having ‘good knowledge and attitude’ based on their responses to some selected questions, this could introduce an interpretation bias about the overall knowledge and attitude of students towards TB. Questions related to TB attitudes may have been subject to social desirability bias and led to participants to under-report negative attitude towards TB.

## Conclusions

The present study revealed non-health science university students in eastern Ethiopia lack important TB-related knowledge and that the majority of them have unacceptable attitudes towards TB. Knowledge and attitudes towards TB varied by university and knowledge was lower among those from rural areas. University administrators, together with the Federal Ministry of Health and other stakeholders should consider providing regular health education programs and advertising to improve TB knowledge and attitudes on university campuses.

## Supplementary information

**Additional file 1 Table S2.** Tuberculosis knowledge about cause, transmission, treatment and prevention among university students, eastern Ethiopia.

**Additional file 2 Table S4.** Attitude towards tuberculosis patients among University students, eastern Ethiopia.

**Additional file 3.** Questionnaire for assessment of TB knowledge and attitude among university students.

## Data Availability

All data generated or analyzed during this study are included in this published article and its supplementary information file.

## Abbreviations

DDU: Dire Dawa University
DOTS: Directly Observed Treatment Short Course program
HU: Haramaya University
JJU: Jigjiga University
KAP: Knowledge, attitude and Practices
SNNP: Southern Nations and Nationality People

## References

[1] WHO 2019. Global tuberculosis report 2019. Geneva: World Health Organization; 2018. Licence: CC BY-NC-SA 3.0 IGO. available at http://apps.who.int/iris.

[2] Esmael A, Ali I, Agonafir M, Desale A, Yaregal Z, Desta K (2013). Assessment of Patients’ Knowledge, Attitude, and Practice Regarding Pulmonary Tuberculosis in Eastern Amhara Regional State, Ethiopia: Cross-Sectional Study. Am. J. Trop. Med. Hyg.

[3] Gelaw M, Genebo T, Dejene A, Lemma E, Eyob G (2001). Attitude and social consequences of tuberculosis in Addis Ababa, Ethiopia. East Afr Med J.

[4] Teran C, Gorena U, Gonzalez B (2015). Knowledge, attitudes and practices on HIV/AIDS and prevalence of HIV in the general population of Sucre. Braz J Infect Dis.

[5] Francisco G, Jay C, Daniel B, Larry J (2001). Treating cardiovascular disease with antimicrobial agents: a survey of knowledge, attitudes, and practices among physicians in the United States. Clin Infect Dis.

[6] Jurcev-Savicevic A, Popovic-Grle S, Milovac S, Ivcevic I, Vukasovic M, Viali V (2008). Tuberculosis knowledge among patients in out-patient settings in Split, Croatia. Int J Tuberc Lung Dis.

[7] Mekonnen A, Collins JM, Aseffa A, Ameni G, Petros B (2018). The Prevalence of Pulmonary Tuberculosis among Students in three Eastern Ethiopia Universities. Int J tuber Lung Dis.

[8] Mekonnen A (2016). and Petros Beyene. Burden of Tuberculosis among students in two Ethiopian Universities. Ethiop Med J.

[9] Federal Democratic Republic of Ethiopia, Federal Ministry of Education, Education Management Information System (EMIS) and Information Communications Technology Directorate. Education Statistics Annual Abstract 2015/16. Addis Ababa, Ethiopia: MoE, 2017. http://www.moe.gov.et/documents/20182/0/ESSAþ2008þE.Cþ%282015-2016þG. C%29/9ae19287-21c9-431b-b53a-d4edfb5fb997?version¼1.0.

[10] Mitike G, Kebede D, Yeneneh H (1997). Prevalence of antituberculosis drug resistance in Harar Tuberculosis Centre, Ethiopia. East Afr Med J.

[11] Masud R, Abu S, Reazul K, Nurul I, Rafiqul I, Tunku K et al. Assessment of knowledge regarding tuberculosis among non-medical university students in Bangladesh: a cross-sectional study. BMC Public Health. 2015; 15:716. 10.1186/s12889-015-2071-0 PMC4517636 26215721

[12] Adane K, Spigt M, Johanna L, Dorscheidt Noortje D, Abera SF, Dinant GJ (2017). Tuberculosis knowledge, attitudes, and practices among northern Ethiopian prisoners: Implications for TB control efforts. PLoS ONE.

[13] World Health Organization. Advocacy, communication and social mobilization for TB control. A guide to developing knowledge, attitude, and practice surveys suiza: WHO; 2008. whqlibdoc.who.int/publications/2008/9789241596176_eng.pdf.

[14] Hassan A, Olukolade R, Ogbuji Q, Afolabi S, Okwuonye L, Kusimo O, Osho J, Osinowo K, and Ladipo O. Knowledge about Tuberculosis: A Precursor to Effective TB Control—Findings from a Follow-Up National KAP Study on Tuberculosis among Nigerians. Tuberc Res Treat. 2017. doi: 10.1155/2017/6309092. 10.1155/2017/6309092 PMC5624149 29075531

[15] Sagbakken M, Frich JC and Bjune GA. Perception and management of tuberculosis symptoms in Addis Ababa, Ethiopia. Qual Health Res. 2008; 18: 1356–66. 10.1177/1049732308322596 18703818

[16] Khalil A, Abdalrahim M. Knowledge, attitudes, and practices towards prevention and early detection of chronic kidney disease. Int Nurs Rev. 2014;61:237–45. 10.1111/inr.12085 24571391

[17] Calderón C, Urizar D, Blázquez C, Ferreras B, Rubio O, Montrull F et al. Knowledge, attitudes and practices on HIV/AIDS and prevalence of HIV in the general population of Sucre. Braz J Infect Dis. 2015; 19:369–75. 10.1016/j.bjid.2015.04.002 PMC9427503 26001978

[18] Kadam S, Shaikh A, Bhati P, Singh S and Dhakne S. Assessment of College student’s awareness about tuberculosis in Ahmednagar. Int. J. Res. Dev. Pharm. L. Sci. 2012/13;2(1): 244–247.

[19] Gele A, Bjune G and Abebe F. Pastoralism and delay in diagnosis of TB in Ethiopia. BMC Public Health. 2009; 9:5 doi:10.1186/1471-2458-9-5. 10.1186/1471-2458-9-5 PMC2628652 19128498

