# Supplementary material for: Tuberculosis knowledge and attitude among non-health science university students needs attention: a cross-sectional study in three Ethiopian universities
Source: BMC Public Health. 2020 May 6;20:631. doi: 10.1186/s12889-020-08788-1 (PMC7203974; doi:10.1186/s12889-020-08788-1)
Supplement: Supplementary file 1 — Additional file 1 Table S2. Tuberculosis knowledge about cause, transmission, treatment and prevention among university students, eastern Ethiopia. [file 12889_2020_8788_MOESM1_ESM.pdf]

Table 2 Tuberculosis knowledge about cause, transmission, treatment and prevention among non-health science university students, eastern Ethiopia

Variables	Frequency n (%)
**Is TB communicable**	
No	70 (4.1)
Yes	1390 (80.8)
No idea	260 (15.1)
**Disease seriousness**	
Very serious	1100 (64.0)
Somewhat serious	370 (21.5)
Not serious	148 (8.6)
No Idea	102 (5.9)
C**auses**	
Don’t know	104 (6.0)
Bad luck/curse	88 (5.1)
Cold wind	481 (28.0)
Smoking	192 (11.2)
Viruses	214 (12.4)
Bacteria	555 (32.3)
Others*	86 (5.0)
**Transmission mode**	
Don’t know	81 (4.7)

Through the air when the infected Person coughs or sneezes	1439 (83.7)
Through eating from the same plate	83 (4.8)
Through touching items in public places (doorknobs, handles in transportation)	105 (6.1)
Through handshakes	12 (.7)
**Can TB be cured?**	
No	15 (.9)
Yes, Completely	1312 (76.3)
Yes, Partially	267 (15.5)
No idea	126 (7.3)
**The best means of treatment**	
Don’t Know	56 (3.3)
Herbal Remedies	67 (3.9)
Homemade Remedies	68 (4.0)
Prayer	87 (5.1)
Holy water	28 (1.6)
Modern medicine	1414 (82.2)
**Know about DOTS program**[¥]	
No	831 (48.3)
Yes	583 (33.9)
**Risks if treatment is not taken**	
Don’t know	197 (11.5)
Infect others	542 (31.5)

Loss weight	155 (9.0)
Develops severe health problem	224 (13.0)
No effect	47 (2.7)
Death	555 (32.3)
**Risks if treatment is not completed**	
Don’t know	282 (16.4)
Drug resistance	163 (9.5)
Relapse	115 (6.7)
Inability to cure infection	504 (29.3)
No effect	17 (1.0)
Death	639 (37.2)
**Means of prevention**	
Do not know	121 (7.0)
Covering mouth and nose when coughing or sneezing	1008 (58.6)
Avoid sharing dishes	107 (6.2)
Washing hands after touching items in public places	61 (3.5)
Closing windows at home	98 (5.7)
Closing windows during public transportation (bus, taxi)	78 (4.5)
By vaccination	115 (6.7)
By isolating TB patients	78 (4.5)
Others	54 (3.1)
**Know about anti-TB vaccine**	
No	391 (22.7)

Yes	993 (57.7)
No idea	336 (19.5)
**Heard of MDR-TB**	
No	884 (51.4)
Yes	836 (48.6)
**Heard of Latent TB**	
No	1540 (89.5)
Yes	180 (10.5)

*Others: Demon, Spoiled soil, Malnutrition, poor hygiene; [¥]total is: All students

DOTS: Directly Observed Treatment Short Course program
