# Supplementary material for: Tuberculosis knowledge and attitude among non-health science university students needs attention: a cross-sectional study in three Ethiopian universities
Source: BMC Public Health. 2020 May 6;20:631. doi: 10.1186/s12889-020-08788-1 (PMC7203974; doi:10.1186/s12889-020-08788-1)
Supplement: Supplementary file 2 — Additional file 2 Table S4. Attitude towards tuberculosis patients among University students, eastern Ethiopia. [file 12889_2020_8788_MOESM2_ESM.pdf]

Table 4 Attitude towards tuberculosis patients among non-health science university students, eastern Ethiopia

Variables	Frequency n (%)
**How TB cases are regarded in his/her community**	
Not sure what to say	256 (14.9)
No reaction	217 (12.6)
Most people reject him/her	138 (8.0)
Most people are friendly, but they generally try to avoid him/her	628 (36.5)
The community mostly supports and helps him/her	481 (28.0)
**Feeling if he/she is diagnosed with TB**	
Do not know	185 (10.8)
Fear	294 (17.1)
Shame	50 (2.9)
Sadness or hopelessness	142 (8.3)
Seek immediate medication	1049 (61.0)
**Would you hide it if you had TB?**	
No	1160 (67.4)
Yes	344 (20.0)
Do not know	216 (12.6)
**Perception about TB affecting his/her social relationship, if he/she is infected**	
No	868 (50.5)

Yes	555 (32.3)
Do not know	297 (17.3)
**What to do if he/she had symptoms of TB**	
Do not know	167 (9.7)
Go to the Clinic in this campus	1000 (58.1)
Go to private clinics outside this campus	185 (10.8)
Go to government health facilities outside this campus	225 (13.1)
Others*	143 (8.3%)
**Perception about the cost of TB treatment in Ethiopia**	
Do not know	512 (29.8)
Free of charge	793 (46.1)
Reasonably priced	206 (12.0)
Expensive	209 (12.2)
**Think HIV positive people should be concerned about TB?**	
No	380 (22.1)
Yes	1216 (70.7)
Don’t know	124 (7.2)
**Feeling about people with TB disease**	
No particular feeling	386 (22.4)
Feel compassion and desire to help	739 (43.0)
Feel compassion, but tend to stay away from these people	382 (22.2)
Fear them because they may infect	213 (12.4)

*Others include: Go to pharmacy to buy anti TB drugs, pursue other self-treatment options (herbs, etc.); go to traditional healer; go back to parents leaving the University
