# Supplementary material for: Tuberculosis knowledge and attitude among non-health science university students needs attention: a cross-sectional study in three Ethiopian universities
Source: BMC Public Health. 2020 May 6;20:631. doi: 10.1186/s12889-020-08788-1 (PMC7203974; doi:10.1186/s12889-020-08788-1)
Supplement: Supplementary file 3 — Additional file 3. Questionnaire for assessment of TB knowledge and attitude among university students. [file 12889_2020_8788_MOESM3_ESM.pdf]

Annex . Questionnaire for Knowledge and Attitude on TB among non-health science university students, eastern Ethiopia

Higher Education Institution________________ Building No.________
Student name_________________________Code:_____________
Student's Mobile Phone No._______________________

Part-I Socio Demographic Characteristics			
S No.	Questions to be asked	Proposed response/s	Coded response
01	Gender	1. Male	0
		2. Female	1
02	Age in Years	___________	
03	Address before joining higher education Institution (by region)	1. Tigray region	0
		2. Afar region	1
		3. Amhara region	2
		4. Oromia region	3
		5. Somali region	4
		6. Benishangul-Gumuz region	5
		7. Gambela region	6
		8. Harari region	7
		9. Southern Nations, Nationalities, & peoples' region	8
		10. Addis Ababa City Administration	9
		11. Dire Dawa City Administration	10
		12. Others	11
04	Residence before joining higher education Institution	1. Urban	0
		2. Rural	1
05	Year of student in the University	1. Year-1	0
		2. Year-2	1
		3. Year-3	2
		4 Year-4	3
		5. Year-5	4
		6. Year-6	5
		7. Above Year-6	6
06	Religion	1. Orthodox	0
		2. Muslim	1
		3. Protestant	2
		4. Catholic	3
		5. Others/Specify______________	4
**Part-II TB Knowledge and awareness**			
07	Have you heard about TB	No	0
		Yes	1
08	If Yes, what was your source of information about TB	Don't know	0
		Radio	1
		Friends	2
		Primary health care center	3

		Family	4
		Newspaper	5
		'Life experience'	6
		Television	7
09	Is TB communicable disease?	No	0
		Yes	1
		I have no Idea	2
10	In your opinion, how serious disease is TB? (chose only one)	Very serious	0
		Somewhat serious	1
		Not very serious	2
		I have no idea	3
11	What do you think are causes of TB?	I don't know	0
		Bad luck/curse	1
		Poor hygiene	2
		Demon	3
		Cold wind	4
		Spoiled soil (soil with a bad odor)	5
		Smoking	6
		Malnutrition	7
		Viruses	8
		Bacteria	9
		Others (specify) ……………..	10
12	What organs of human body can be affected by TB	I have no Idea	0
		Bones	1
		Kidney	2
		Uterus	3
		Abdomen	4
		Lungs	5
		Others/Specify	6
13	How can a person infected with TB transmit it to another person?	Don't know	0
		Through the air when the infected Person coughs or sneezes	1
		Through eating from the same plate	2
		Through touching items in public places (doorknobs, handles in transportation)	3
		Through handshakes	4
		Others (specify) --------------------	5
14	In your opinion, who can be infected with TB? (Check all that are relevant)	I have no idea	0
		Only people living with HIV/AIDS	1
		Only children	2
		Only women	3
		Only old people	4
		Only poor people	5

		Only homeless people	6
		Only alcoholics	7
		Anybody	8
		Others/specify	9
15	Which of the following are signs or symptoms of TB affecting the lungs? (Please check all that are mentioned)	Do not know	0
		Fever	1
		Hemoptysis/Coughing up blood	2
		Night sweats	3
		Loss of appetite	4
		Chest pain	5
		Loss of Weight	6
		General weakness	7
		Cough>2weeks	8
		Others/specify	9
16	How can one prevent infection with TB? (Please check all that are mentioned.)	Do not know	0
		Covering mouth and nose when coughing or sneezing	1
		Avoid sharing dishes	2
		Washing hands after touching items in public places	3
		Closing windows at home	4
		Closing windows during public transportation (bus, taxi)	5
		Through good nutrition	6
		By prayer	7
		By vaccination	8
		By isolating TB patients	9
		Avoid shaking hands	10
		Others (specify)	11
17	Can TB be cured?	No	0
		Yes, completely	1
		Yes, Partially	2
		I have no idea	3
		Yes	1
18	What is the best treatment for someone with TB? (chose only one)	Don’t Know	0
		Herbal Remedies	1
		Homemade Remedies	2
		Prayer	3

		Holy water	4
		Specific drugs given at health facilities/Modern medicine	5
19	If "modern medicine" for Q no. 18; have you heard about TB-DOTS (Directly Observed Treatment, Short course) program	No	0
		Yes	1
20	If "modern medicine" for Q no. 18, how long is the TB treatment?	I have no idea	0
		Less than 3 months	1
		3 months	2
		6 months	3
		9 months	4
		1 year	5
21	Is there a vaccine for TB?	No	0
		Yes	1
		I have no idea	2
22	Which of the following are risks if a person with TB does not take treatment? (Please check all that are mentioned)	I don't know	0
		Infects others	1
		Losses weight	2
		Develops sever health problems	3
		No effect	4
		Death	5
		Others (specify) ___________________	6
23	Which of the following are risks if a person with TB does not finish a full course of treatment? (Please check all that are mentioned.)	I don't know	0
		Drug resistance	1
		Relapse	2
		Inability to cure infection	3
		No effect	4
		Death	5
24	Have you heard about drug resistant TB/ MDR?	No	0
		Yes	1
25	If yes, how serious is MDR-TB	I have no idea	0
		Not very serious	1
		Somewhat serious	2
		Very serious	3
26	Have you heard of latent TB?	No	0
		Yes	1
	**Part-III. Attitude and practices towards TB**		
27	In your community, how is a person who has TB usually regarded/treated?	Not sure what I would feel	0
		No reaction	1
		Most people reject him/her	2
		Most people are friendly, but they generally try to avoid him/her	3

		The community mostly supports and helps him/her	4
		Other/specify	5
28	Which of the following best describes how you would feel if you were diagnosed with TB? (chose only one)	I do not know	0
		Fear	1
		Surprise	2
		Shame	3
		Sadness or hopelessness	4
		Seek immediate medication	5
29	If you had TB, would you hide it from others?	No	0
		Yes	1
		I do not know	2
30	If you had TB, do you think it would affect any of your social relationships?	No	0
		Yes	1
		I do not know	2
31	Are you afraid of being infected with TB? (chose only one)	No	0
		Yes	1
32	If you or someone living in your dormitory develops TB, do you encourage other students in your dormitory to get tested?	No	0
		Yes	1
33	What would you do if you thought you had symptoms of TB? (chose only one)	Do not know	0
		Go to the Clinic in this campus	1
		Go to private clinics outside this campus	2
		Go to government health facilities outside this campus	3
		Go to pharmacy to buy anti TB drugs	4
		Go to traditional healer	5
		Pursue other self-treatment options (herbs, etc.)	6
		Go back to my parents, leaving the University	7
		Others (specify) ____________________	8
34	If you would not seek care for TB, what is the reason? (Please check all that apply)	Not sure where to go	0
		Cost	1
		Difficulties with transportation/distance to health facilities outside this campus	2
		Do not trust medical workers in this campus	3
		Do not like attitude of medical workers in this campus	4
		Others (specify) ____________________	5
35	If you would not go to a health	When treatment on my own does not work	0

	clinic after developing symptoms of TB, at what point would you seek treatment? (chose only one)	When symptoms that look like TB signs last for 3–4 weeks.	1
		As soon as I realize that my symptoms might be related to TB	2
		I would not go to the doctor/I would be cured as TB is self-limited disease	3
36	How would you rate the cost of TB treatment in Ethiopia? (chose only one)	Do not know	0
		It is free of charge	1
		It is reasonably priced	2
		It is somewhat/moderately expensive	3
		It is very expensive	4
37	Do you believe the risk of TB is higher at Universities or in the community? (chose only one)	Do not know	0
		In the University	1
		In the community	2
38	If you think the risk of TB is higher at universities, what is the reason? (Please check all that apply)	Don't know	0
		Inadequate access to food and health services	1
		Overcrowding	2
		Poor ventilation	3
		Easily acquiring TB infection	4
		Others (specify) __________________	5
39	Do you think that HIV positive people should be concerned about TB?	No, Why	0
		Yes, Why	1
		Do not know	2
40	Which statement is closest to your feeling about people with TB disease?	"I have no particular feeling."	0
		"I feel compassion and desire to help."	1
		"I feel compassion, but I tend to stay away from these people."	2
		"It is their problem and I cannot get TB."	3
		"I fear them because they may infect me."	4
		Other (please explain): _______________	

Name of Data Collector___________________________ Date___________________Sign____________

Thanks for your participation!
